# Supplementary material for: Chromothripsis during telomere crisis is independent of NHEJ, and consistent with a replicative origin
Source: Genome Res. 2019 May;29(5):737–49. doi: 10.1101/gr.240705.118 (PMC6499312; doi:10.1101/gr.240705.118)
Supplement: Supplemental Material [file supp_gr.240705.118_Supplemental_file_1.zip › contigs/annotated_contigs/DB57/contig.2.DB57_length_280_mean_cov_18.4678571429.docx]

**DB57_length_280_mean_cov_18.4678571429**

```
TCTGGAAAGGTGGCTTAATGTATGGGCTCTAGAATAAGAAAAACTTAGACCCTTATCTTGACTCTGCTTCTTATCAGCTATTTGAACGT
 >chr7:141222173-141222329 - E=1e-82
GGCCAAGGCTCTGTTTTTATTGGTAAAACAGAGATAATAAGACAGAGGTCATAGGGTTATTGGGAGC|GTAGCGTCTGTGGTGGGCTCT
                                                                     >chr7:157234700-157
TCCGGGCGGCGCCTGGCGTAGCGTCTGGGGTGGGCTGTTCCACGCGGGCGCCTGACGTAGCGTCTTTGGTGGGCTCTTCTGGGCGGCGC
234824 + E=2e-57
CTGGCGTAGCGTCT
```
